# Infarct of the Right Basal Ganglia in a Male Spinal Cord Injury Patient: Adverse Effect of Autonomic Dysreflexia

**DOI:** 10.1100/tsw.2011.55

**Published:** 2011-03-22

**Authors:** Subramanian Vaidyanathan, Bakul M. Soni, Gurpreet Singh, Peter L. Hughes, Kamesh Pulya, Tun Oo

**Affiliations:** ^1^ Regional Spinal Injuries Centre, District General Hospital, Southport, Merseyside, UK; ^2^ Department of Urology, District General Hospital, Southport, Merseyside, UK; ^3^ Department of Radiology, District General Hospital, Southport, Merseyside, UK; ^4^ Department of Cardiology, District General Hospital, Southport, Merseyside, UK

**Keywords:** spinal cord injury, autonomic dysreflexia, basal ganglia, paraplegia, urinary bladder, intermittent catheterisation

## Abstract

Autonomic dysreflexia is a clinical emergency that occurs in individuals with spinal cord injury at level T-6 and above. We present a 58-year-old male patient with paraplegia who developed a severe, recurrent, throbbing headache during the night, which was relieved by emptying the urinary bladder by intermittent catheterisation. As this person continued to get episodes of severe headache for more than 6 months, computed tomography (CT) of the brain was performed. CT revealed an infarct measuring 1.2 cm in the right basal ganglia. In order to control involuntary detrusor contractions, the patient was prescribed propiverine hydrochloride 15 mg four times a day. The alpha-adrenoceptor blocking drug doxazosin was used to reduce the severity of autonomic dysreflexia. Following 4 weeks of treatment with propiverine and doxazosin, the headache subsided completely. We learned from this case that bladder spasms in individuals with spinal cord injury can lead to severe, recurrent episodes of autonomic dysreflexia that, in turn, can predispose to vascular complications in the brain. Therefore, it is important to take appropriate steps to control bladder spasms and thereby prevent recurrent episodes of autonomic dysreflexia. Intermittent catheterisations along with an alpha-adrenoceptor blocking drug (doxazosin) and an antimuscarinic drug (propiverine hydrochloride) helped this individual to control autonomic dysreflexia, triggered by bladder spasms during the night.

## INTRODUCTION

Autonomic dysreflexia is a clinical emergency that occurs in individuals with spinal cord injury at level T-6 and above. An episode of autonomic dysreflexia is characterised by acute elevation of arterial blood pressure, bradycardia, severe headache, profuse sweating, flushing, and piloerection above the level of the spinal cord injury. Although these represent typical symptoms of autonomic dysreflexia, the symptoms can vary dramatically. The severity of symptoms does not correlate with the severity of hypertension and autonomic dysreflexia can occur without any symptoms at all (i.e., silent autonomic dysreflexia).

Persistent or malignant autonomic dysreflexia is uncommon in individuals with spinal cord injury[1] and is usually caused by noxious stimuli that cannot be removed promptly, e.g., somatic pain, abdominal distension. When the inciting cause for autonomic dysreflexia is not amenable for treatment, the severity of dysreflexic episodes can be decreased by pharmacotherapy with an alpha-adrenergic blocking agent (doxazosin) and sustained-release nifedipine[2]. However, it should be emphasised that pharmacological intervention must be closely monitored due to the lability of blood pressure in these individuals with spinal cord injury. Blood pressure may be easily overcorrected and hypotension may be produced by pharmacotherapy for autonomic dysreflexia.

We present a gentleman with paraplegia who developed a severe, recurrent, throbbing headache during the night, which was relieved by emptying the urinary bladder by intermittent catheterisation. This individual continued to get severe headaches during the night for more than 6 months, and the headaches would subside after he performed self-catheterisation. When this person presented with such a long duration of recurrent episodes of headache, investigations were performed to look for cerebrovascular complications of autonomic dysreflexia, e.g., intracranial bleed. Computed tomography (CT) of the brain revealed an infarct measuring 1.2 cm in the right basal ganglia.

## CASE PRESENTATION

A British Caucasian male, born in 1952, had been suffering from ankylosing spondylitis. He fell down in 2006 and sustained a fracture of T-6 with incomplete paraplegia (American Spinal Injury Association [ASIA] impairment scale category – D). He was managing his bladder by intermittent self-catheterisation. In 2008, this patient sustained another fall while he was in the shower. He had fracture through T-11/12 disc space. Posterior spinal internal fixation was carried out, extending from T-7 to L-3 and entailing a pedicular screw system and rods.

In February 2010, this individual started waking up during the night almost every 2 h with severe headaches. He would then perform self-catheterisation and the headaches would go away. He was taking the following medications:

- Bisacodyl 5 mg by mouth
- Senna syrup 20 ml
- Bisacodyl rectal solution 1.5 ml per rectum
- Oxybutynin modified-release 10 mg once a day
- Peppermint oil capsule 0.2 ml, one, three times a day

The patient described the headaches as most horrendous and sickening. He continued to wake up with intense headaches every night. After he emptied his urinary bladder by intermittent catheterisation, the headaches went away completely. He noticed blurring of vision as well when he woke up with the severe headaches during the night. When he did not have the headaches, his blood pressure was 134/89 mmHg. In August 2010, he started developing heaviness in his chest in addition to the throbbing headaches that woke him from sleep. The combination of throbbing headache and heaviness in his chest was frightening, and he sought urgent medical care from the spinal unit.

The clinical impression was autonomic dysreflexia, triggered by bladder spasms, which seemed to occur predominantly during the night. In order to control involuntary detrusor contractions, the patient was advised to take propiverine hydrochloride 15 mg, four times a day. The alpha-adrenoceptor blocking drug doxazosin was also prescribed initially in a dose of 1 mg to be taken at night; the dose of doxazosin was increased to 2 mg after 7 days. Doxazosin would control adrenergic overactivity during autonomic dysreflexia.

After 2 weeks, the intensity and frequency of the headaches decreased considerably. When he started getting the headaches, the intensity was 9 on a scale of 0 to 10, but after taking propiverine and doxazosin, the severity of the headaches diminished to 3 or 4. Similarly, the frequency of the headaches also decreased. Whereas 6 months ago he would get up with a severe, throbbing headache almost every 2 h during the night, after he started taking propiverine and doxazosin, he woke up with a headache only once during the night. Following 4 weeks of treatment with the alpha-adrenoceptor blocking drug doxazosin and the antimuscarinic drug propiverine, the headaches subsided completely. The patient did not develop side effects to doxazosin, such as dizziness or fainting, when he sat up. His blood pressure while sitting up was 135/82 mmHg, heart rate was 83 per minute, and oxygen saturation was 98%.

Ultrasound examination revealed that the right kidney measured 13 cm with multiple focal cortical scarring in the mid pole. There was no hydronephrosis. The left kidney measured 12.5 cm with a parenchymal depth of 2 cm. There was a calculus in the mid pole calyx measuring 1.2 cm. There was no hydronephrosis. No calculus was seen in the urinary bladder (Fig. 1).

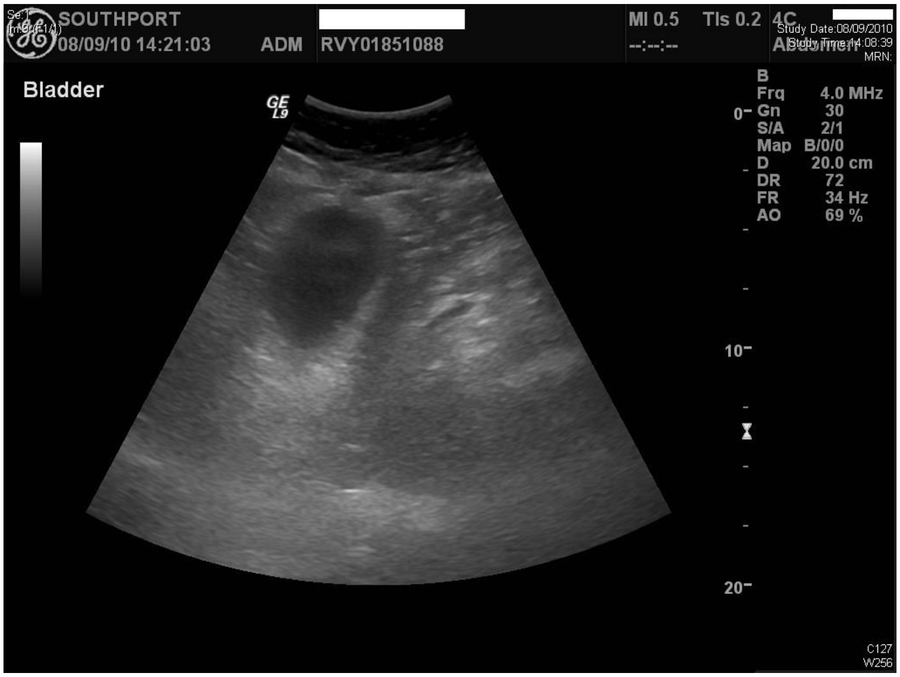

**FIGURE 1.** Ultrasound image of the urinary bladder, performed on 08 September 2010, showed no calculi; bladder was underfilled.

While this patient was taking doxazosin 2 mg and propiverine hydrochloride 15 mg, four times a day, urodynamics were performed. Urodynamics showed an initial residue of about 50 ml, the bladder neck was open, there was no vesicoureteric reflex, and there was no leakage through the bladder neck all through the filling. There was no evidence of detrusor overactivity. When the urinary bladder was filled to about 250 ml, the patient started to feel a bit heavy headed. His blood pressure then was 130/70. Filling of the urinary bladder was continued to about 400 ml; the patient's blood pressure remained at 130/70. Thus, urodynamics reassured the patient and the health professionals that the combination of doxazosin and propiverine hydrochloride had controlled the autonomic dysreflexia, the bladder capacity had increased to 400 ml, and there was no increase in blood pressure even after filling the bladder to 400 ml.

Since the patient experienced heaviness in the chest when he developed the pounding headaches, a 24-h ECG was performed. The dominant rhythm was sinus, but frequent ventricular ectopics as isolated beats (total 2826) with two couplets and 50 trigeminy cycles were seen. ECG showed mild impairment of the left ventricular systolic function and sclerotic aortic valve. Left and right atria were of normal size.

As this individual with spinal cord injury had been getting recurrent episodes of autonomic dysreflexia for more than 6 months, CT of the brain was performed to look for cerebrovascular complications of autonomic dysreflexia, such as intracranial bleed. CT of the brain revealed a 1.2-cm area of low attenuation in the right basal ganglia in keeping with an infarct (Fig. 2). There was no evidence of intracranial bleed. There was no mass effect. When a small infarct in the right basal ganglia was detected, the patient's neurology was reassessed. He had not experienced any functionally significant change in motor function of the upper limbs. There was no involuntary movement of the upper limbs, head, and neck. However, it should be remembered that this patient had severe ankylosing spondylitis and any involuntary movement of the head would be virtually impossible. However, the patient's wife had noticed subtle personality changes in him for the past month; he would get angry and sometimes forgetful.

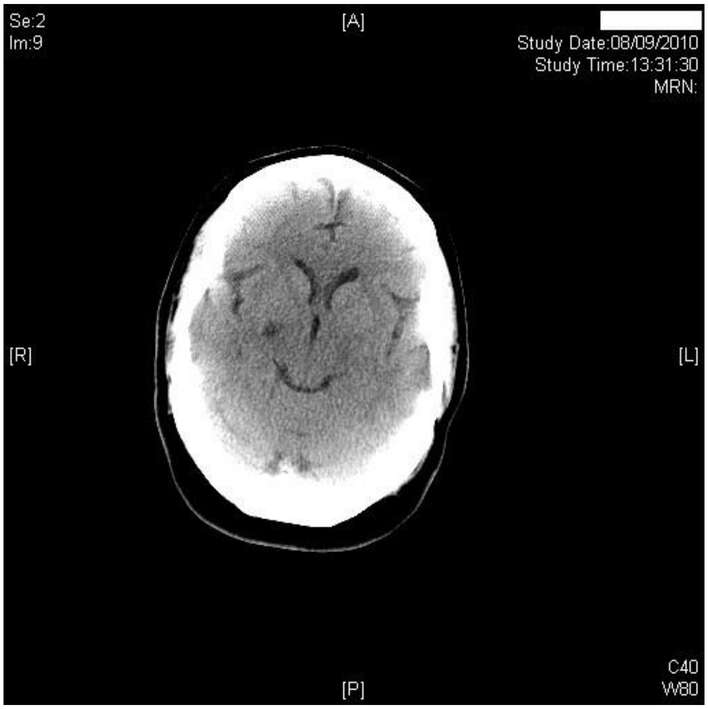

**FIGURE 2.** CT of the brain, performed on 08 September 2010, revealed a 1.2-cm area of low attenuation noted in the right basal ganglia, which would be in keeping with an infarct. There was no evidence of an intracerebral bleed and no mass effect.

A duplex study of the carotid arteries was performed. Both common carotid arteries appeared free from significant plaque with no turbulent flow or high velocities. Bifurcation and entrance to the internal carotid artery demonstrated mild plaque on both sides. B-mode imaging suggested a stenosis of <50% in the right and left internal carotid arteries. The external carotid artery on both sides had no significant plaque or high-velocity flow. The subclavian artery was seen on both sides and appeared to have normal multiphasic flow.

This spinal cord injury patient was prescribed atorvastatin 10 mg, as CT revealed an infarct in the right basal ganglia. He was advised to take aspirin 75 mg once a day. However, he said that he had tried taking aspirin in the past and it made him very poorly; therefore, aspirin was not prescribed to him.

This patient was advised follow-up in the spinal unit clinic every month with the aim to (1) monitor and prevent occurrence of autonomic dysreflexia, (2) improve quality of life related to bladder management, and (3) track changes related to infarct of the basal ganglia.

## DISCUSSION

The development of autonomic dysreflexia correlates with aberrant sprouting of peptidergic afferent fibres into the spinal cord below the injury. In particular, sprouting of nerve growth factor–responsive afferent fibres has been shown to have a major influence on dysreflexia, perhaps by amplifying the activation of disinhibited sympathetic neurons[3]. Physicians and individuals with spinal cord injury should be aware that, due to changes in the nervous system over time following injury, autonomic dysreflexia can develop or change in severity over time.

Autonomic dysreflexia can be a hypertensive episode in persons with spinal cord injury. An increase in blood pressure is induced by exaggerated sympathetic activity, which is thought to be mediated by the alpha-adrenergic system[4]. Therefore, alpha-adrenoceptor antagonists have been a rational first choice for control of autonomic dysreflexia; nevertheless, calcium channel blockers are primarily used to decrease blood pressure during an acute episode of autonomic dysreflexia. We prescribed the alpha-adrenoceptor blocking drug doxazosin to our patient in order to reduce the severity of dysreflexic episodes. As the primary cause for dysreflexic episodes was involuntary contraction of the urinary bladder (detrusor hyper-reflexia), the patient was advised to take the antimuscarinic drug propiverine hydrochloride, which would reduce involuntary detrusor contractions and increase bladder capacity.

Spinal cord injury causes disruption of descendent pathways from the brain to spinal sympathetic neurons, originating into intermediolateral nuclei of T-1 to L-2 spinal cord segments. Loss of supraspinal control over the sympathetic nervous system results in reduced overall sympathetic activity below the level of the injury and unopposed parasympathetic outflow through the intact vagal nerve. Thus, spinal cord injury may lead to significant cardiac dysfunction, mostly in patients with cervical or high thoracic injury. Cardiac dysrhythmias, especially bradycardia, and, rarely, cardiac arrest, or tachyarrhythmias, and hypotension may occur soon after spinal cord injury[5]. In the chronic phase of spinal cord injury, autonomic dysreflexia can occur when a significant increase in visceral sympathetic activity with coronary artery constriction can result in myocardial ischemia, even in the absence of coronary artery disease[6]. Persons with spinal cord injury who develop autonomic dysreflexia may sustain complications of the central nervous system, such as intracranial haemorrhage, posterior leukoencephalopathy[7], and cerebral vasoconstriction syndrome[8]. A search in PubMed revealed no report of infarct in the basal ganglia following autonomic dysreflexia in persons with spinal cord injury. Our patient developed severe, recurrent, autonomic dysreflexia and CT of the brain revealed infarct in the right basal ganglia. Edvardsson and Persson[8] described a 32-year-old man with quadriparesis due to traumatic C-5/6 fracture who experienced recurrent thunderclap headaches following autonomic dysreflexia due to a neurogenic bladder and urinary tract infection. Selective catheter cerebral angiography revealed multiple calibre changes in the intracranial blood vessels, thus establishing a diagnosis of reversible cerebral vasoconstriction syndrome due to autonomic dysreflexia. A magnetic resonance imaging (MRI) of the brain after 2 weeks revealed ischaemic changes in the left hemisphere. MRI/CT of the brain after 6 months demonstrated a large infarction in the left hemisphere. We did not perform MRI or angiography of the brain in this patient, as he was frightened of undergoing MRI. It seemed that in another hospital, his scalp was caught by the machine as he went through the scanner. The scalp was about to be ripped off and the machine had to be stopped as an emergency measure. This left a very traumatic experience in our patient who was too frightened to go through the scanner again.

Basal ganglia infarcts are a relatively rare type of lacunar infarct and clinical presentations are subtle. Behavioural symptoms predominate in persons with infarct of the basal ganglia, and these may be combined with other subtle motor and speech abnormalities. The behavioural abnormalities include abulia, apathy, akinesia, amnesia, disinhibition, hemi-neglect, and affective symptoms. Abulia, which is defined as lack of initiative and emotions, slowness, and exhibiting delayed responsiveness and actions, is the most common behavioural abnormality observed, occurring in an estimated 13% of basal ganglia infarcts[8]. Wagner and Begaz[9] state that spouses have a unique insight into their partner's behaviour and their concerns must be taken seriously; cerebrovascular lesions should be considered in the differential diagnosis of these cases and appropriate imaging pursued in order to detect rare cases of basal ganglion stroke. In our case, the patient's spouse had noticed subtle changes in his personality. With humility, we admit that we failed to attach significance to the observations made by the patient's spouse in the beginning. CT showed infarct of the right basal ganglion that would explain subtle personality changes observed by the patient's spouse, but were ignored by health professionals.

This patient with spinal cord injury and autonomic dysreflexia was managed by intermittent catheterisations along with a combination of an antimuscarinic drug and alpha-adrenoceptor blocking agent. However, there are other options for management of this case. For example, an individual with spinal cord injury and severely reduced bladder capacity, clearly impairing quality of life, may be considered an external catheter drainage for night-time management as an alternative to medication. Of course, such individuals need to be monitored closely by urodynamic studies at regular intervals. Botulinum toxin type A injected into the detrusor has been shown to be a safe and efficacious treatment for spinal cord injured patients with refractory detrusor overactivity[10]; this effect is maintained at 26 weeks postinjection.

This case also illustrates the need for education of individuals with spinal cord injury and their caregivers on early recognition of the symptoms of autonomic dysreflexia. It is unfortunate that the patient was allowed to suffer for 6 months prior to seeking urgent medical attention. McGillivray and associates[11] concluded that promotion of knowledge about recognizing and managing autonomic dysreflexia might help to reduce the risk of cardiac and cerebrovascular disease in individuals with spinal cord injury.

## CONCLUSION

We learned from this case that bladder spasms can lead to severe, recurrent episodes of autonomic dysreflexia that, in turn, may predispose to vascular complications in the brain. This individual with spinal cord injury developed infarct of the right basal ganglion, which is irreversible. Therefore, it is important that appropriate steps are taken promptly to control bladder spasms and thereby prevent recurrent episodes of autonomic dysreflexia. Intermittent catheterisations along with a combination of an alpha-adrenoceptor blocking drug (doxazosin) and an antimuscarinic drug (propiverine hydrochloride) helped to control involuntary detrusor contractions and autonomic dysreflexia, which was triggered by bladder spasms.

Much of the literature on autonomic dysreflexia in individuals with spinal cord injury focuses on alleviation of the root cause of the blood pressure elevations (i.e., bladder and/or bowel emptying, loosening of tight clothing, repositioning, etc.), which would be of little benefit to an individual who frequently experiences autonomic dysreflexia and may demonstrate unappreciated neuroanatomical evidence of infarct. Unfortunately, autonomic dysreflexia is often untreated until the signs are clinically evident (pounding headache, chest compression, sweating, etc.), but the findings presented herein suggest that subclinical evidence may relate to cognitive impairments and personality changes. This case presentation might be the first to illuminate the important, yet unappreciated, notion that although clinical evidence is not really observable, basal ganglion infarct may result from autonomic dysreflexia, which would impair the lives of these individuals; thus autonomic dysreflexia should be treated prophylactically.

## ACKNOWLEDGEMENT

The authors are grateful to Ms. Jacquie McGarry, Senior Territory Manager, Hollister Continence Care Division, Rectory Court, 2 Broad Street, Wokingham, Berks, U.K. for the valuable help towards payment of open access fee for this paper.
